# 

**Published:** 1851-08

**Authors:** 


### TO CORRESPONDENTS.

We have to beg the indulgence of our correspondents. Several valuable communicatiens are on file for an early number. We solicit papers from our friends in relation to the epidemics now prevalent in the country. Cholera is appearing, from time to time, in various places, and dysentery is reported to be fatal in many parts.

### *Receipts of Medical Journal for July*, 1851.

| | |
|---|---|
| Dr. J. Lundy, Salem, Ky., - - - - - | $5 00 |
| W. J. Routh, Cassville, Mo., - - - | 5 00 |
| A. H. Hawkins, Waverly, Miss., - - - | 5 00 |
| B. L. Hatch. Aberdeen, Miss. - - - | 5 00 |
| Albany Med. College, Albany, N. Y., - - - | 5 00 |
| Dr. R. Hodgen, Campbellsville, Ky., - - - | 5 00 |
| W. J. Holmes, McCallister's ⋈ Roads, Tenn., - | 5 00 |
| E. W. Harris, Cape Girardeau, Mo., - - | 10 00 |

The WESTERN JOURNAL OF MEDICINE and SURGERY is published monthly by the undersigued, at the corner of Main and Fifth streets, Louisville, at $5 per annum, payable in advance. Each number contains 96 pages, making two volumes in the year of more than 550 pages each.

Contributions to its pages by the Physicians of the Valley of the Mississippi addressed to the Editors, are respectfully solicited.

Letters on business, to be addressed, postage paid, to the publishers.

PRENTICE & CO.

